# Profile and determinants of acute stroke outcome: a prospective cohort study in a tertiary healthcare facility in Makurdi, North Central Nigeria

**DOI:** 10.11604/pamj.2025.52.189.49928

**Published:** 2025-12-30

**Authors:** Emmanuel Iwuozo, Samuel Utim, Innocent Okwori, Richard Bolade, Msonter Anzaa, Elochukwu Aroh

**Affiliations:** 1 Neurology Unit, Medicine Department, Benue State University Teaching Hospital, Makurdi, Benue State, Nigeria,; 2 Department of Epidemiology and Community Health, Benue State University Teaching Hospital, Makurdi, Benue State, Nigeria,; 3 Department of Anatomy, College of Medicine, Enugu State University of Science and Technology, Enugu, Nigeria

**Keywords:** Profile, stroke, stroke outcome, stroke mortality, determinants

## Abstract

**Introduction:**

stroke remains a major cause of morbidity and mortality, particularly in low-resource settings. This study assessed the profile and determinants of outcome in acute stroke patients in a tertiary health care facility in Makurdi, North Central Nigeria.

**Methods:**

a prospective cohort study was conducted between April 2021 and August 2022 at the Benue State University Teaching Hospital, Makurdi. Consecutively admitted patients (≥18 years) with acute stroke were recruited and assessed using a structured questionnaire and the Modified Rankin Scale (mRS). Descriptive statistics were used to compute the mean and standard deviation for age, while multivariable regression analysis was done to determine factors associated with mortality.

**Results:**

out of 105 patients, 80.0% (n=84) were aged ≥50 years (mean = 60 ± 13), and 58.1% (n=61) were male. Ischaemic stroke was more frequent, 78.1% (n=82), than hemorrhagic stroke, 21.9% (n=23). Hypertension, 74.3% (n=78), and diabetes, 36.2% (n=38) were the leading risk factors. The case fatality rate was 37.1% (n=39), while 55.3% (n=58) improved and were discharged. A good outcome (mRS 0-3) was achieved in 46.7% (n=49) of the subjects. Multivariate analysis revealed that vomiting (aOR: 11.55, 95% CI 1.65-80.71; p=0.014), unsteady gait (aOR: 12.93, 95% CI 1.27-132.22; p=0.031), and cognitive impairment (aOR: 7.42, 95% CI 1.46-37.87; p=0.016) markedly increased the likelihood of death.

**Conclusion:**

the majority of stroke patients in Makurdi suffered an ischaemic stroke type, and had hypertension as the main risk factor, with a case fatality rate of 37.1%. Furthermore, vomiting, unsteady gait, and cognitive impairment were strongly associated with death as an outcome.

## Introduction

Stroke, which can be of the ischaemic (85%) or haemorrhagic (15%) type, is a major public health problem that occurs when the blood supply to a part of the brain is interrupted or reduced, depriving brain tissue of oxygen and nutrients, with potential brain tissue death [1,2]. Stroke ranks as the second leading cause of mortality among non-communicable diseases (NCDs). It has been reported that low- and middle-income countries (LMICs), especially in Africa, account for about 90% of the burden of stroke, and Nigeria has a prevalence of 26 per 100,000 person-years [3-7].

The largest landmark global case-control study on stroke risk factors, INTERSTROKE, revealed diabetes mellitus, cigarette smoking, physical inactivity, and dietary and psychosocial issues as common risk factors among Africans [4]. Owolabi *et al*. conducted the largest study on stroke in Africa (SIREN), which explored the dominant modifiable risk factors for stroke in Ghana and Nigeria [5]. They reported 11 potentially modifiable risk factors associated with stroke occurrence in decreasing order of magnitude by population-attributable risks (PAR): hypertension, dyslipidemia, regular meat consumption, elevated waist-to-hip ratio, diabetes, low green leafy vegetable consumption, stress, added salt at the table, cardiac disease, physical inactivity, and current use of cigarettes. They opined that these 11 factors collectively accounted for 98.2% of PAR associated with stroke [5].

Stroke is a common neurological emergency that requires prompt diagnosis and early initiation of appropriate, evidence-based treatment to improve the outcome; however, a reversal from this is observed in LMICs [8-10]. Poor stroke outcome in many resource-limited countries could be attributable to a dearth of trained personnel in acute stroke care, unavailability of thrombolytic agents, and generally the lack of an organised stroke unit/well-structured rehabilitation programs [11,12]. Some studies identified unfavourable outcomes with subtype, admission duration, elevated glycated haemoglobin in diabetics, loss of consciousness at presentation, presence of atrial fibrillation, and cardiac disease. Others are HIV infection and high National Institute of Health Stroke Severity Scale (NIHSS) scores [13,14]. In a multicenter prospective cohort study by the SIREN team, patient-level determinants of stroke fatality were low vegetable consumption, high systolic blood pressure, stroke lesion volume more than 30 cm^3^, high NIHSS score, elevated intracranial pressure, and aspiration pneumonia [15]. Hence, the overall objective of this study is to assess the profile and determinants of outcome in acute stroke patients in a tertiary health care facility in Makurdi, North Central Nigeria.

## Methods

**Study design and setting:** this is a prospective hospital-based cohort study. It was conducted between April 2021 and August 2022 at the Benue State University Teaching Hospital, located in Makurdi, North Central Nigeria. It serves as one of two tertiary hospitals in the State with eighteen specialist departments and a total staff strength of 1,700. It is a top referral center for a population of 4.2 million people comprising different tribes (Tiv, Idoma, Igede, and Etulo, etc.), religions (Christians/Muslims, etc.), and occupations (civil servants, farmers, traders, etc.), according to the 2006 national population census [16,17].

**Study population:** these comprised patients with acute stroke admitted to the emergency unit and medical wards of the Benue State University Teaching Hospital. The study included consecutively admitted patients with a diagnosis of acute stroke presenting within 1 month of the event. They also had to be at least 18 years old and diagnosed to have had a stroke based on brain computed tomography (CT) scan or magnetic resonance imaging findings. They were excluded from the study if they had stroke-like states, like a brain tumour, brain abscess, or transient ischaemic attack.

**Data collection:** a structured questionnaire and the Modified Rankin Scale were utilised in the recruitment of participants. The Modified Rankin Scale (mRS) was used at the point of enrollment, and Fisher's exact test was used to test the relationship between categorical variables. For this study, trained clinicians, after obtaining appropriate consent from the patients/proxies, meticulously entered the confidential data obtained into the questionnaire.

**Definitions:** the structured questionnaire assessed socio-demographic factors like age, sex, stroke type, stroke risk factors, body mass index, occupation, and education, etc. and clinical characteristics like stroke duration, stroke risk factors, stroke type, etc. The mRS is a six-point disability scale with scores ranging from 0 to 6, with higher scores denoting worse clinical outcomes [18]. Scores of 0-3 were categorised as good outcome (no disability), scores of 4-5 as handicapped, and scores of 6 as dead [19]. The outcome was categorised into dead, left against medical advice, improved and discharged, and referred.

**Statistical analysis:** data entry and statistical analysis were done using the statistical package for social sciences (SPSS) software (version 25; SPSS, Chicago, IL, USA). Descriptive statistics were used to compute the mean and standard deviation for age. Fisher's exact test was used to test the relationship between categorical variables, as more than 20% of the cells had expected counts less than 5. Univariable multinomial logistic regression (reference: discharged/improved) was performed. The relative risk ratio (RRR), 95% confidence interval (CI) and the p-value of each variable were noted. Variables with p<0.05 and 95% CI not crossing 1 were considered significant predictors of the outcome, and all variables with p<0.10 were selected for multivariable analysis. These included: limb weakness, loss of consciousness, confusion, vomiting, unsteady gait, dizziness, aphasia, and cognitive impairment. Multivariable multinomial logistic regression was performed to model the 4-category outcome (dead, discharged/improved, left against medical advice, referred) with discharged/improved as the reference category. Forward stepwise selection based on likelihood ratio tests (entry p<0.05, removal p<0.10) was used to build the model. Outcome categories were compared to discharged/improved. The model provided a significant improvement in fit over the null model (X^2^=62.83, df=24, p<0.001) implying that the included predictors substantially explained variation in outcomes. It explained 53% of the variance (Naelkerke R2=0.534). Multicollinearity was assessed using variance inflation factors (VIF), and all VIFs were <5, indicating no substantial correlations among predictors.

**Ethical considerations:** ethical approval was obtained from the Health Research Ethical Committee (HREC) of the institution with number BSUTH/MKD/HREC/2020/020 before commencement of the study. Informed consent was obtained from all participants or their proxies, with confidentiality of all data acquired ensured.

## Results

**General characteristics of study participants:** the majority of the subjects, 80.0% (n=84), were aged 50 years and above (mean = 60 ± 13), while 20.0% (n=21) were below 50 years. A little over half of the subjects, 58.1% (n=61), were males. while the rest, 41.9% (n=44), were females. Most of the subjects, 38.1% (n=40), were self-employed; some 34.3% (n=36) were unemployed, and 27.6% (n=29) were employed. More than half of the participants, 52.4% (n=55), had a tertiary level of education; 19.0% (n=20) had primary, 14.3% (n=15) had secondary, and 14.3% (n=15) had no formal education. More than half of the subjects, 58.1% (n=61), presented within 10 days of stroke symptom onset. The majority of them, 87.6% (n=92), spent about 10 days on admission. Over half of the subjects, 51.3% (n=54), had left hemispheric stroke, while some had both, 4.8% (n=5). The commonest form of stroke was ischaemic in 78.1% (n=82), while 21.9% (n=23) had hemorrhagic stroke. The leading risk factor for stroke among the participants was hypertension, 74.3% (n=78), while the least risk factor among them was sickle cell, 1.0% (n=1). The commonest symptom among the subjects was limb weakness, 70.5% (n=74), while none had visual loss. The commonest CT diagnosis was infarction, 64.8% (n=68), while the least common was subarachnoid haemorrhage, 1.0% (n=1). The details are as seen in Table 1 and Table 1.1.

**Table 1:** baseline characteristics of participants (n=105)

Patient socio-demographic characteristics	Frequency	Percent (%)
**Age (years)**		
< 50	21	20.0
≥50	84	80.0
**Sex**		
Male	61	58.1
Female	44	41.9
**Occupation**		
Formal	29	27.6
Informal	40	38.1
Unemployed	36	34.3
**Education**		
None	15	14.3
Primary	20	19.0
Secondary	15	14.3
Tertiary	55	52.4
**Patient clinical characteristics**		
**Stroke duration (days)**		
1-10	61	58.1
11-20	24	22.9
21-30	20	19.0
**Duration on admission (days)**		
1-10	92	87.6
11-20	11	10.4
>20	2	2.0
**Stroke hemisphere**		
Right	46	43.9
Left	54	51.3
Both	5	4.8
**Handedness**		
Right	104	99.0
Left	1	1.0
**Stroke type**		
Infarction	82	78.1
Hemorrhagic	23	21.9
**Risk factors**		
Hypertension	78	74.3
Diabetes	38	36.2
Obesity	14	13.3
Dyslipidemia	14	13.3

**Table 1.1:** baseline characteristics of participants (n=105)

Clinical characteristics	Frequency (n)	Percent (%)
**Risk factors**		
Smoking	7	6.7
Sickle cell	1	1.0
Alcohol	13	12.4
Heart disease	6	5.7
Family history	2	1.9
Previous stroke	23	21.9
TIA	4	3.8
**Symptom**		
Facial asymmetry	50	47.6
Limb weakness	74	70.5
Loss of consciousness	47	44.8
Confusion	23	21.9
Seizures	13	12.4
Vomiting	20	19.4
Unsteady gait	13	12.4
Dizziness	5	4.8
Visual loss	0	0.0
Abnormal sensation	4	3.8
Aphasia	38	36.2
Cognitive impairment	19	18.1
**CT diagnosis**		
Infarction	68	64.8
Intracranial hemorrhage	19	18.1
Subarachnoid hemorrhage	1	1.0
Normal	17	16.1
**Patient treatment and outcome**		
**Treatment**		
IV fluid	69	65.7
IV mannitol	20	19.0
Antiplatelet	85	81.0
Antihypertensive	23	21.9
Antibiotics	48	45.7
Anticoagulants	17	16.2
Statins	73	69.5
Physiotherapy	52	49.5
Speech therapy	21	20.0
**Stroke outcome**		
Dead	39	37.1
Left against medical advice	4	3.8
Improved/discharged	58	55.3
Referred	4	3.8

**Figure 1:**
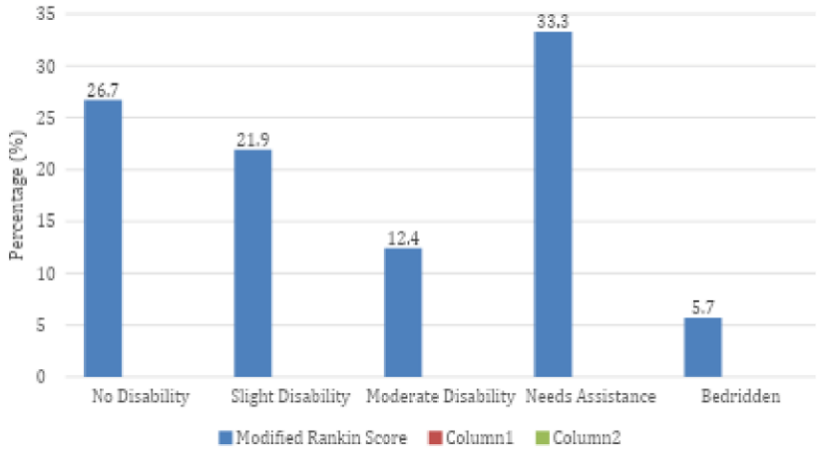
modified Rankin score of participants at recruitment (n=105)

**Treatment and outcome of participants:** most of them received antiplatelets 82.0% (n=86), statins 69.5% (n=73), and intravenous fluids 65.7% (n=69). The least received modality of treatment was anticoagulation, 16.2% (n=17). More than half, 55.2% (n=58), of the participants improved and were discharged, while 37.1% (n=39) died, 3.8% (n=4) were referred, and 3.8% (n=4) left against medical advice. Regarding the modified Rankin Score (mRS) at recruitment: 26.7% (n=28) of the stroke patients had no significant disability, 21.9% (n=23) had slight disability, 12.4% (n=13) had moderate disability, 33.3% (n=35) needed assistance, and 5.7% (n=6) were bedridden. The details are as seen in Table 1, Table 1.1 and Figure 1, respectively.

**Relationship between sociodemographic and clinical characteristics and stroke outcome:** among the socio-demographic characteristics, only education (P=.03) had a significant relationship with the outcome of treatment among the subjects at a P≤.05 level of significance. Among the clinical factors, obesity (P=.01), limb weakness (P=.001), loss of consciousness (P=.01), confusion (P=.02), vomiting (P=.002), unsteady gait (P=.004), aphasia (P=.02), and cognitive impairment (P=.002) were significantly associated with the outcome. The details are as seen in Table 2 and Table 2.1.

**Table 2:** relationship between patients’ characteristics and stroke outcome

Patient characteristics	Stroke outcome				Fisher's exact test p-value
	Dead	Left against medical advice	Improved/discharged	Referred	
**Sex**					.743
Male	21	2	36	2	
Female	18	2	22	2	
**Age**					.787
<50	6	1	13	1	
≥50	33	3	45	3	
**Occupation**					.452
Formal	10	3	14	2	
Informal	16	0	23	1	
Unemployed	13	1	21	1	
**Education**					.033
Primary	14	0	6	0	
Secondary	5	0	10	0	
Tertiary	13	4	35	3	
None	7	0	7	1	
**Stroke duration (days)**					.071
1-10	26	2	32	1	
11-20	8	1	14	1	
21-30	5	1	12	2	
**Duration on admission (days)**					.125
1-10	33	4	51	4	
11-20	6	0	5	0	
21-30	0	0	2	0	
**Handedness**					1.000
Right	39	4	57	4	
Left	0	0	1	0	
**Stroke hemisphere**					.794
Right	16	1	25	1	
Left	20	3	28	3	
Both	3	0	2	0	
**Hypertension**					.564
Yes	31	3	42	2	
No	8	1	16	2	
**Diabetes**					.098
Yes	17	3	16	2	
No	22	1	42	2	
**Obesity**					.014
Yes	4	1	6	3	
No	35	3	52	1	
**Dyslipidemia**					.283
Yes	3	1	9	1	
No	36	3	49	3	
**Smoking**					.500
Yes	2	0	4	1	
No	37	4	54	3	
**Sickle cell**					.448
Yes	1	0	0	0	
No	38	4	58	4	

**Table 2.1:** relationship between patients’ characteristics and stroke outcome

Patient characteristics	Stroke outcome				Fisher’s exact test p-value
	Dead	Left against medical advice	Improved/discharged	Referred	
**Alcohol**					.788
Yes	5	1	7	0	
No	34	3	51	4	
**Heart disease**					.434
Yes	2	1	3	0	
No	37	3	55	4	
**Family history of stroke**					.586
Yes	0	0	2	0	
No	39	4	56	4	
**Previous stroke**					.216
Yes	12	0	11	0	
No	25	4	47	4	
**TIA**					.744
Yes	1	0	3	0	
No	38	4	55	4	
**Facial asymmetry**					.401
Yes	15	2	30	3	
No	24	2	28	1	
**Limb weakness**					.001
Yes	22	1	49	2	
No	17	3	9	2	
**Loss of consciousness**					.006
Yes	25	2	18	2	
No	14	2	40	2	
**Confusion**					.018
Yes	15	0	8	0	
No	24	4	58	4	
**Seizures**					.253
Yes	6	1	5	1	
No	33	3	53	3	
**Vomiting**					.002
Yes	13	2	4	1	
No	26	2	54	3	
**Unsteady gait**					.004
Yes	11	0	2	0	
No	28	4	56	4	
**Dizziness**					.093
Yes	0	0	4	1	
No	39	4	54	3	
**Abnormal sensation**					.744
Yes	1	0	3	0	
No	38	4	55	4	
**Aphasia**					.024
Yes	7	2	27	2	
No	31	2	31	2	
**Cognitive impairment**					.002
Yes	14	1	4	0	
No	25	3	54	4	

**Determinants of stroke outcome:** the variables that reached significance in the univariable analysis included: limb weakness, loss of consciousness, confusion, vomiting, unsteady gait, dizziness, aphasia, and cognitive impairment. Vomiting (aOR: 6.35, 95% CI 1.30-31.8; P=.023), unsteady gait (aOR: 6.61, 95% CI 1.02-42.69; P=.047), and cognitive impairment (aOR: 7.02, 95% CI 1.49-32.99; P=.014) were significantly associated with high odds of death, while limb weakness (aOR: 0.06, 95% CI 0.004-0.99; P=.049) was significantly associated with lower odds of leaving against medical advice. The details are as seen in Table 3.

**Table 3:** determinants of stroke outcome

Variable	Unadjusted OR (95% CI)a	P-value	Adjusted OR (95% CI)a	P-value
**Death**				
Limb weakness	0.24 (0.09-0.62)	.003	0.51 (0.15-1.74)	.28
Loss of consciousness	3.97 (1.68-9.37)	.002	1.98 (0.65-6.00)	.23
Confusion	3.91 (1.46-10.48)	.007	1.51 (0.37-6.6.16)	.57
Vomiting	6.75 (2.00-22.74)	.002	6.35 (1.30-31.08)	.02
Unsteady gait	11.00 (2.28-56.06)	.003	6.61 (1.02-42.69)	.047
Aphasia	0.26 (0.10-0.68)	.006	0.59 (0.18-1.96)	.39
Cognitive impairment	7.56 (2.26-25.31)	.001	7.02 (1.49-32.99)	.014
**Left against medical advice**				
Limb weakness	0.06 (0.01-0.66)	.021	0.06 (0.004-0.99)	.049
Loss of consciousness	2.22 (0.29-17.05)	.442	0.51 (0.03-8.36)	.63
Vomiting	13.5 (1.49-122.75)	.021	10.91 (0.73-162.43)	.08
Aphasia	1.15 (0.15-8.71)	.89	2.84 (0.24-33.98)	.41
Cognitive impairment	4.50 (0.38-53.77)	.24	8.68 (0.26-285.23)	.23
**Referred**				
Limb weakness	0.18 (0.02-1.48)	.11	0.19 (0.02-2.15)	.18
Loss of consciousness	2.22 (0.29-17.05)	.442	1.21 (0.12-12.75)	.87
Vomiting	4.50 (0.38-53.77)	.235	2.04 (0.11-37.48)	.63
Aphasia	1.15 (0.15-8.71)	.89	1.54 (0.16-15.22)	.71

a = OR is the odds ratio, and CI is the confidence interval

## Discussion

The objective of this study was to assess the profile and determinants of outcomes among patients with acute stroke presenting to a tertiary health facility in Makurdi, North Central Nigeria. The most salient findings were that ischaemic stroke predominated, hypertension was the leading risk factor, and the case fatality rate was high at 37.1%. Moreover, vomiting, unsteady gait, and cognitive impairment independently predicted mortality, while education was the only sociodemographic factor significantly associated with outcome. The predominance of ischaemic stroke (78.1%) in this study is consistent with global and regional epidemiology, where ischaemic stroke accounts for 70-85% of all stroke cases [1,2]. Similar findings have been reported in other Nigerian and African cohorts, including those from Southeast Nigeria and Ethiopia, where ischaemic stroke was more common than hemorrhagic stroke [13,20,21]. Hypertension emerged as the most frequent risk factor, affecting 74.3% of the sample, which aligns with findings from INTERSTROKE and SIREN, both of which identified hypertension as the dominant modifiable contributor to stroke risk in African populations [4,5]. This underscores the persistent challenge of hypertension control in sub-Saharan Africa and the need for strengthened primary prevention strategies [21-23].

The case fatality rate of 37.1% in our cohort is considerably high and mirrors reports from other low-resource settings, where mortality ranges from 21 to 45% [20,23,24]. Multiple factors may explain this elevated mortality. First, over half of patients presented after 10 days of symptom onset, significantly outside the window for acute interventions, including thrombolysis or stroke unit care—interventions known to improve outcomes [8,25]. Second, the hospital lacks an organised stroke unit, a deficit documented across many African facilities and associated with poor outcomes [9-12,25-27]. Third, a substantial proportion of patients had severe deficits on admission, as reflected by high mRS scores; one-third required assistance, and about 6% were bedridden.

A major contribution of this study is the identification of vomiting, unsteady gait, and cognitive impairment as independent predictors of death. Vomiting (aOR 6.35) is recognised as a warning sign of raised intracranial pressure, often associated with large infarcts or intracerebral haemorrhage, both of which carry high mortality [15,28]. Similarly, unsteady gait (aOR 6.61) may indicate cerebellar involvement or extensive posterior circulation stroke, with established associations with rapid deterioration if untreated [28]. Cognitive impairment (aOR 7.02) may reflect global cerebral dysfunction or diffuse ischemic injury, and previous studies have shown that altered mental status strongly correlates with mortality in acute stroke [15,24]. These findings highlight the importance of early neurological assessment and close monitoring of patients presenting with these clinical features. Interestingly, limb weakness—although common—was not an independent predictor of mortality but was significantly associated with lower odds of leaving against medical advice. One possible explanation is that patients with overt neurological deficits may be more likely to remain hospitalised due to the obvious need for care, whereas those with milder or fluctuating symptoms may opt for premature discharge, a pattern previously documented in LMIC settings [20]. The finding that education level was the only sociodemographic predictor of outcome aligns with evidence that higher education improves health-seeking behaviour, treatment adherence, and understanding of disease processes [29]. Individuals with higher educational attainment may present earlier, consent to recommended treatments, and participate more actively in rehabilitation.

The implication of these findings is significant for stroke care in North Central Nigeria and comparable settings. Early recognition of high-risk clinical features—particularly vomiting, unsteady gait, and cognitive impairment—should guide triage decisions and resource allocation, allowing rapid escalation of care. The high case fatality rate underscores the urgent need for organised stroke units, improved ambulance and referral systems, and the availability of acute interventions such as thrombolysis and neurosurgical support. Strengthening hypertension control programs at the community level remains essential, given the high prevalence of this risk factor [25,26].

This study has notable strengths. Its prospective design reduces recall bias, and the use of a standardised outcome measure (mRS) improves comparability with global stroke research [18,19]. The multivariable modelling provides robust insights into independent predictors of mortality, adding to the limited literature from North Central Nigeria. However, some limitations must be acknowledged. Being a single-center study, the findings may not be generalizable to the broader Nigerian population. The modest sample size limits the power to detect weaker associations. Neuroimaging follow-up was not uniformly available, preventing assessment of stroke progression or cerebral oedema. Additionally, long-term functional outcomes beyond discharge were not evaluated, although such data would have enriched the understanding of recovery trajectories. Despite these limitations, the study provides important insights into stroke outcomes in a region with limited epidemiological data.

## Conclusion

This study demonstrated that most acute stroke patients in Makurdi suffered ischaemic stroke, with hypertension as the leading risk factor, and a high case fatality rate of 37.1%. Vomiting, unsteady gait, and cognitive impairment were identified as strong independent predictors of death, while education was the only sociodemographic variable associated with outcome. These findings indicate that severe neurological symptoms at presentation substantially worsen prognosis, and delays in presentation and limited stroke care resources likely contribute to poor outcomes. The results highlight the importance of early recognition of high-risk clinical features and underscore the significant burden of hypertension in this population.

### What is known about this topic

- Stroke is a leading cause of morbidity and mortality globally, particularly in low-resource settings such as sub-Saharan Africa;
- Ischaemic stroke is generally more frequent than hemorrhagic stroke, with hypertension as the predominant modifiable risk factor;
- Stroke outcomes are strongly influenced by severity markers such as loss of consciousness, neurological deficits, and comorbid conditions.

### What this study adds

- Categorized stroke outcome into dead, left against medical advice, improved with subsequent discharge, and referred;
- The case fatality rate in Makurdi was high (37.1%), underscoring the urgent need for improved stroke services in North Central Nigeria;
- Vomiting, unsteady gait, and cognitive impairment emerged as strong independent predictors of death, while education was the only socio-demographic factor significantly associated with outcome.
